# Supplementary material for: Structural heterogeneity in the intrinsically disordered RNA polymerase II C-terminal domain
Source: Nat Commun. 2017 May 12;8:15231. doi: 10.1038/ncomms15231 (PMC5437306; doi:10.1038/ncomms15231)
Supplement: Supplementary Information — Supplementary Figures and Supplementary Table [file ncomms15231-s1.pdf]

SUPPLEMENTARY INFORMATION

Structural heterogeneity in the intrinsically disordered RNA polymerase II C-terminal domain.

Bede Portz[1], Feiyue Lu[1,2], Eric B. Gibbs[3], Joshua E. Mayfield[4], M. Rachel[5] Mehaffey, Yan Jessie Zhang[4,6], Jennifer S. Brodbelt[5], Scott A. Showalter[1,3]and David S. Gilmour[1]

[1]Center for Eukaryotic Gene Regulation, Department of Biochemistry and Molecular Biology, [2]The Huck Institutes of Life Sciences. [3]Department of Chemistry, The Pennsylvania State University, University Park, Pennsylvania, 16802, [4]Department of Molecular Biosciences, University of Texas, Austin, Texas 78712, [5]Department of Chemistry, University of Texas, Austin, Texas 78712, [6]Institute for Cellular and Molecular Biology, University of Texas, Austin, Texas 78712

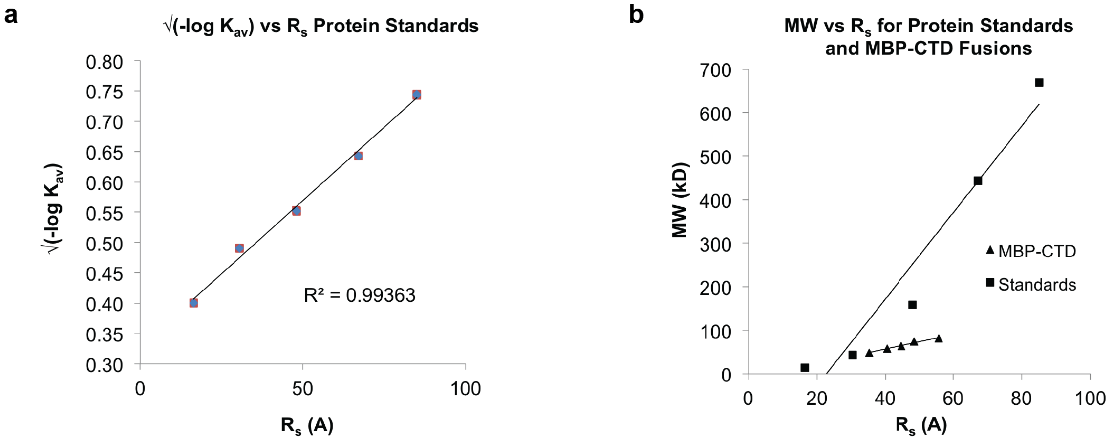

Supplementary Figure 1. (a) Analytical gel filtration standard curve used to determine CTD Stokes Radii (R_S). Standards used, in order of increasing R_s, were RNAseA, ovalbumin, aldolase, apoferritin, and thyroglobulin. (b) Comparison of MW vs R_S for the protein standards (squares) and MBP-CTD fusions (triangles), including MBP alone. The curves converge near MBP (leftmost triangle), but have very different slopes suggesting an extended CTD emanating from the globular MBP. The MBP-CTD fusions have molecular weights (MW) between 58 and 82kD, but migrate as proteins comparable in R_s to aldolase, which has a MW of 158kD, indicating the CTD is more extended than a natively folded protein of similar MW.

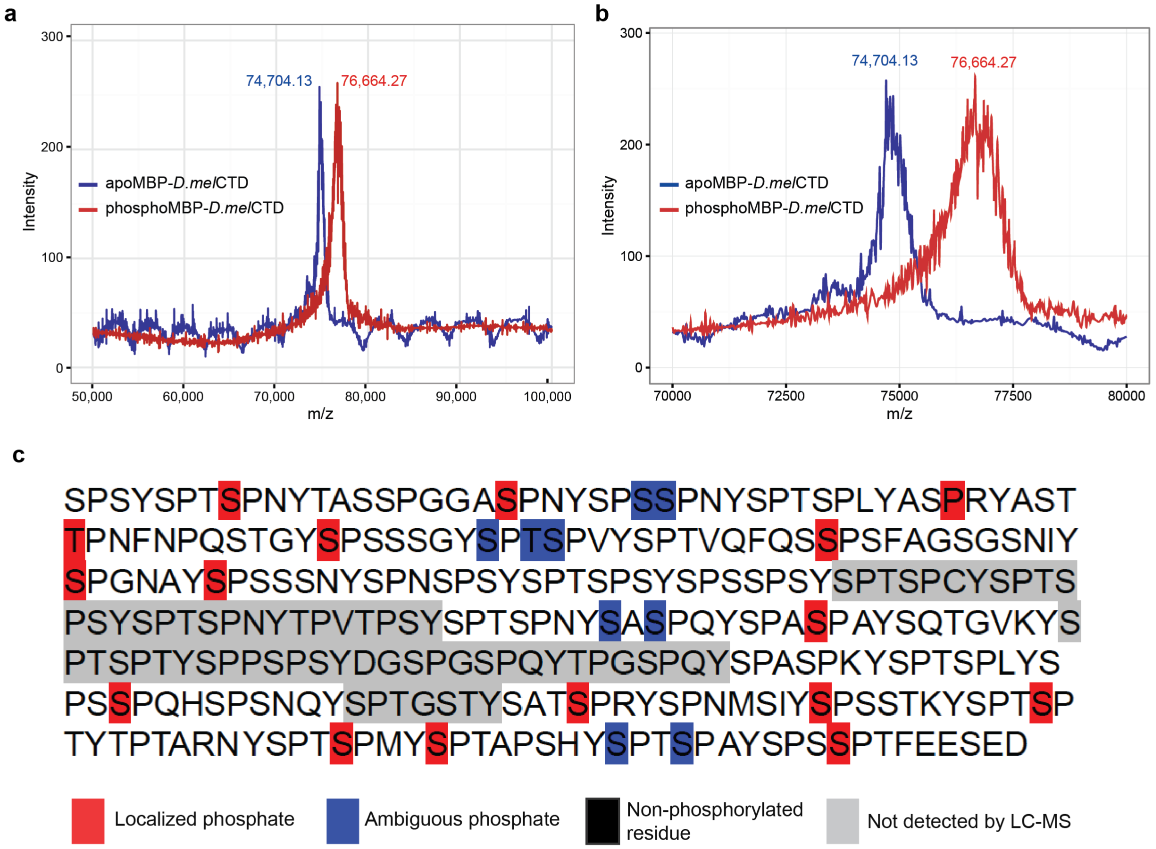

Supplementary Figure 2. (a) MALDI-TOF mass spectra of apo and phospho MBP-*D.mel*CTD fusions indicates an increase in mass of ~1.9 kDa. (b) Zoomed-in section of the spectra from panel a. shows a distribution of phosphorylated species for the phospho MBP-*D.mel*CTD with the average change in mass consistent with the addition of 25 phosphates. (c) Tandem mass spectrometry localizes the sites of phosphate incorporation via LC-HCD-MS analysis of a chymotryptic digest of phosphorylated MBP-D.*mel*CTD. Localized phosphates are highlighted in red, fragments phosphorylated only once, but with the precise location of phosphorylation unable to be precisely determined are highlighted in blue, and regions for which there is insufficient mass spectrometry coverage are shaded in grey. Heptads are phosphorylated only once per repeat, with a preference for S/T in the 5th position, unless the degenerate 5-6 position lacks an SP motif.

a

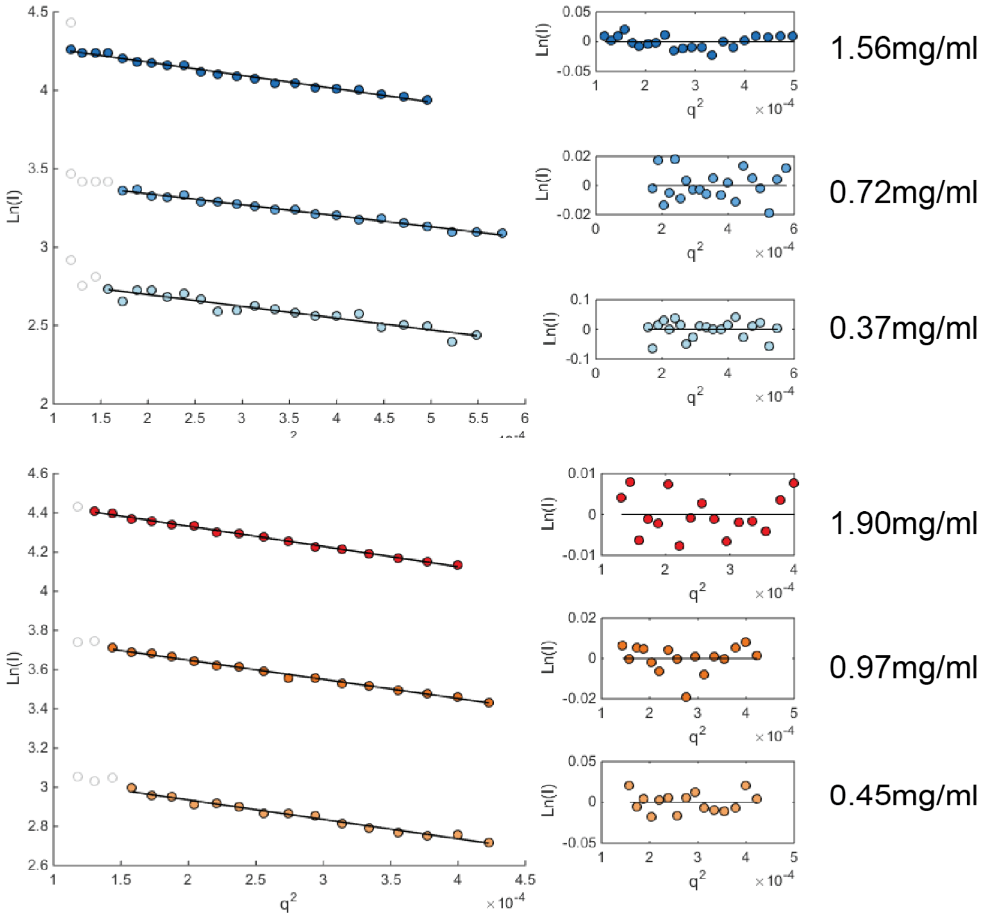

b

	Guinier Fit		
**Protein**	**(mg/ml)**	**MW(kD)**	**R_g(Å)**
**apoCTD**	1.56	79.73	50.69
	0.72	72.74	46.22
	0.37	74.63	47.64
Avg and Std Dev.		**75.7 +/- 3.6**	**48.2 +/- 2.3**
**phosphoCTD**	1.9	78.98	56.02
	0.97	77.04	54.22
	0.45	81.67	54.70
Avg and Std. Dev.		**77.6 +/- 1.6**	**55.0 +/- 0.9**

Supplementary Figure 3. (a) Guinier fits of SAXS curves plotted as ln(I) vs q^2 (left panels) and residuals (right panels) of SAXS data collected at three protein concentrations for the apo (blue data) and phospho (red data) MBP-*D.mel*CTD fusion proteins (b) Summary of Guinier analysis. Calculated MW or Rg are independent of protein concentration as expected for monodisperse samples. Calculated MW is close to that of the apo MBP-*D.mel*CTD fusion, 74.70kD. The Rg of the CTD increases as a result of phosphorylation.

a

D. melanogaster CTD FLAGSTREP TEV site insertion map
|[#] denotes TEV recognition sequence (ENLYFQG)
|[4]SPSYSPTSPNYTASSPGGASPNYSPSSPNYSPTSPLYASPRYASTTPNFNPQ
STGYSPSSSGYSPTSPVY|[3]SPTVQFQSSPSFAGSGSNIYSPGNAYSPSSSNYS
PNSPSYSPTSPSYSPSSPSYSPTSPCYSPTSPSYSPTSPNYTPVTPSY|[2]SPTSP
NYSASPQYSPASPAYSQTGVKYSPTSPTYSPPSPSYDGSPGSPQYTPGSPQY
SPASPKYSPTSPLYSPSSPQHSPSNQYSPTGSTY|[1]SATSPRYSPNMSIYSPSST
KYSPTSPTYTPTARNYSPTSPMYSPTAPSHYSPTSPAYSPSSPTFEESEDDYK
DDDDKWSHPQFEK

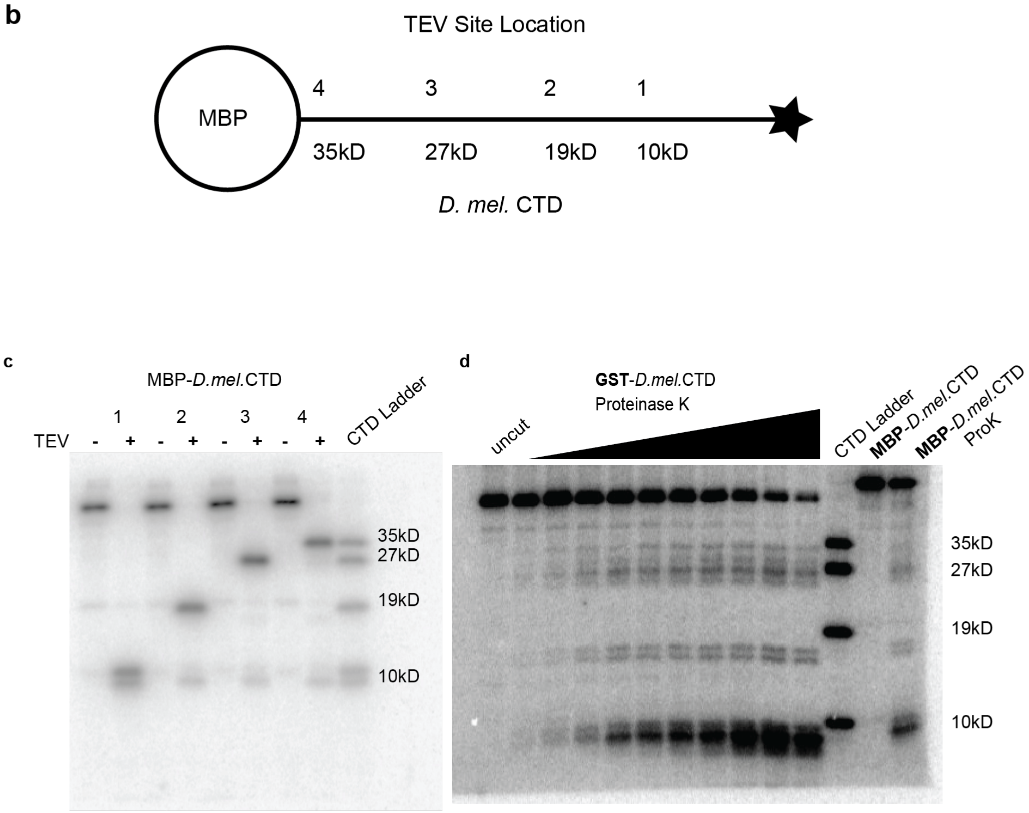

Supplementary Figure 4. (a) Location of TEV protease recognition sites inserted into four separate MBP-*D.mel*CTDFLAGSTREP fusion proteins. Numbered red bars correspond to fusion proteins displayed in panel c. (b) Schematic of radiolabeled TEV product sizes. (c) TEV cleavage generates a CTD ladder ranging in size from the full CTD (35kD) to a 10kD distal fragment. (d) Limited proteolysis of GST-*D.mel*CTD compared to the CTD ladder and MBP-*D.mel*CTD. The discontinuous pattern of proteolysis generated from the GST fusion matches that of the MBP fusion.

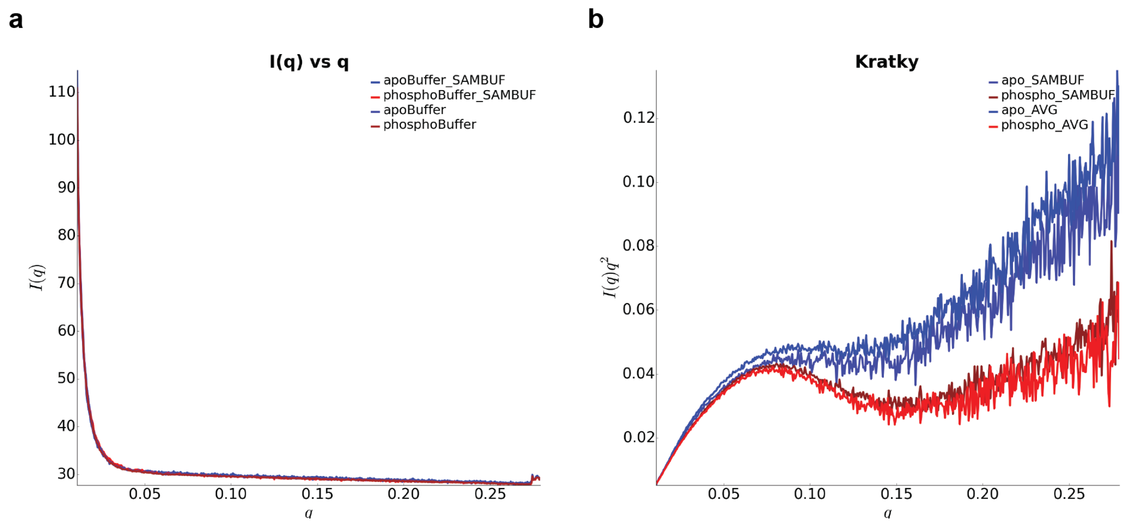

Supplementary Figure 5. (a) I(q) vs q plot of averaged buffer signal used in buffer subtraction of apo and phospho scattering data plotted with buffer scattering curves calculated from the zero concentration extrapolation performed using SAMBUF. The measured and extrapolated buffer scattering curves overlay indicating that we correctly account for and subtract the scattering contribution of the buffer in our analyses. (b) Kratky plots with error bars for averaged scattering curves and for scattering curves derived from zero concentration extrapolation using SAMBUF. Buffer subtracted averaged curves and buffer independent zero concentration extrapolation yield the same result. The agreement between buffer subtracted curves and those derived from buffer independent zero concentration extrapolation validates that the loss of flexibility resulting from phosphorylation is not an artifact of buffer subtraction.

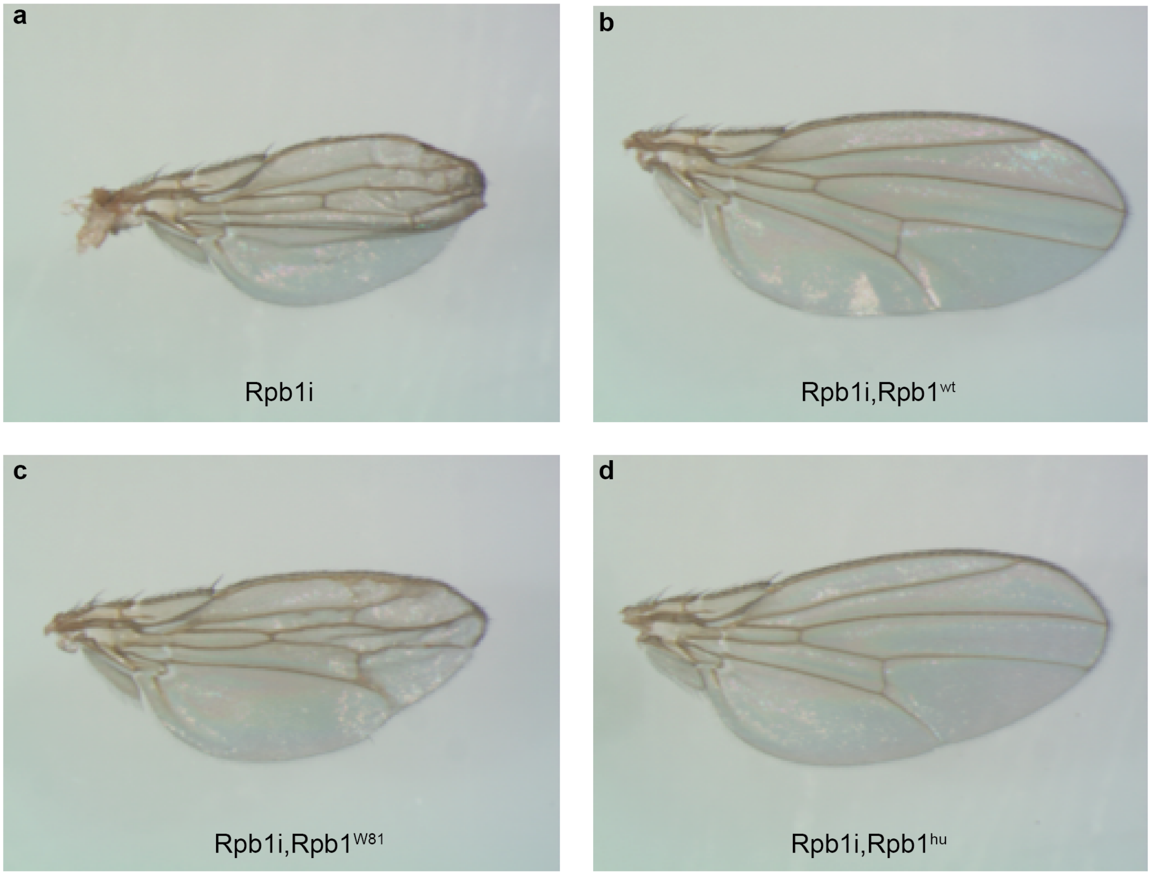

Supplementary Figure 6. Humanized Rpb1 can compensate for the effect of Rpb1 knock down. Photographs of representative wings of offspring from each cross are shown. At least 50 individuals were examined and little phenotypic variation existed among individuals. (a) Rpb1 RNAi (Rpb1i) causes wing undergrowth. (b) Co-expression of RNAi resistant Rpb1 with the Rpb1 RNAi restores normal wing growth. (c) Co-expression of the W81 mutant form of Rpb1 with the Rpb1 RNAi only partially restores wing growth and demonstrates that the assay can detect a range of phenotypes. (d) Co-expression of Rpb1 harboring the human CTD with the Rpb1 RNAi restores wing growth in a manner indistinguishable from wild-type Rpb1.

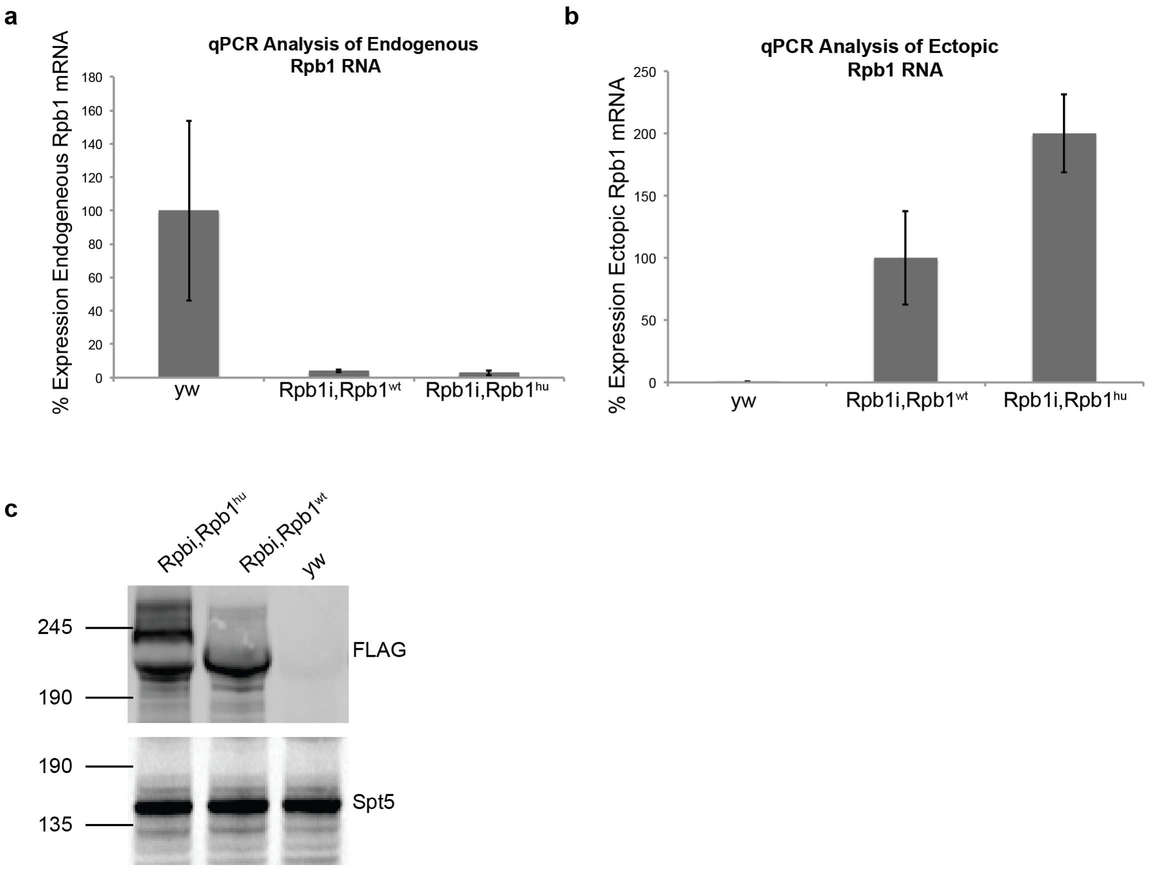

Supplementary Figure 7 The human CTD can function in place of the *Drosophila* CTD *in vivo*. (a) qPCR analysis of RNA isolated from straight winged adult progeny from the Rpb1 RNAi rescue experiments using primers specific for endogenous Rpb1 RNA. Genotypes of the flies are as follows: yw is *ActGAL4/+.* Rpb1i,Rpb1^{wt} is *ActGAL4/+;Rpb1i,Rpb1^{wt}/+*. Rpb1i,Rpb1^{hu} is *ActGAL4/+*; *Rpb1i,Rpb1^{hu}/+* (b) qPCR as in panel a, using primers specific for the ectopically expressed, RNAi resistant Rpb1 RNA. The sequence of the ectopically expressed Rpb1 RNA differs from the endogenous version by a 21 nucleotide region that contains the synonymous mutations which render it resistant to the RNAi. (c) Western blot of late pupae lysates probed with antibody against the FLAG Rpb1 variants or Spt5, which serves as a loading control.

Table 1 Results of p-element complementation crosses.

	FM7c,B,y[31d]/Y; daGal4,UAS Rpb1/+	G0040,w[+],y[+]/Y; daGal4,UAS Rpb1/+	yw/O; daGal4,UAS Rpb1/+
#1 Rpb1[G0040] ♀ X yw; daGal4 ♂	10	0	1
#2 Rpb1[G0040] ♀ X yw; daGal4 ♂	17	0	5
#3 Rpb1[G0040] ♀ X yw; daGal4 ♂	7	0	2
#1 Rpb1[G0040] ♀ X yw; daGal4, UAST-Rpb1 ^{wt} ♂	16	9	2
#2 Rpb1[G0040] ♀ X yw; daGal4, UAST-Rpb1 $^{wt]}$ ♂	11	11	2
#3 Rpb1[G0040] ♀ X yw; daGal4, UAST-Rpb1 ^{wt} ♂	13	4	3
#1 Rpb1[G0040] ♀ X yw; daGal4, UAST-Rpb1$^{hu/}$TM3Sb ♂	6	4	1
#2 Rpb1[G0040] ♀ X yw; daGal4, UAST-Rpb1 $^{hu/}$/TM3Sb ♂	6	4	1
#3 Rpb1[G0040] ♀ X yw; daGal4, UAST-Rpb1 $^{hu/}$]/TM3Sb ♂	5	5	0
